# Secondary Immune Thrombocytopenic Purpura in Renal Cell Cancer: A Paraneoplastic Syndrome

**DOI:** 10.7759/cureus.4398

**Published:** 2019-04-05

**Authors:** Arish Noor, Anam Rehan, Aakash Desai, Corina Iorgoveanu, Jim C Lee

**Affiliations:** 1 Internal Medicine, University of Connecitcut, Hartford, USA; 2 Internal Medicine, University of Connecticut, Hartford, USA; 3 Pathology, Hartford Hospital, Hartford, USA

**Keywords:** paraneoplastic syndrome, immune thrombocytopenic purpura, renal cell cancer

## Abstract

Secondary immune thrombocytopenic purpura (ITP) as a paraneoplastic syndrome has been reported in literature. It is commonly associated with chronic lymphocytic leukemia (CLL) and hodgkins lymphoma. Its association with solid malignancies, especially renal cell cancer is rare, with only a few documented case reports. Treatment usually consisted of targeting the underlying malignancy or utilization of steroid and intravenous immunoglobulin (IVIG) to improve thrombocytopenia. Here, we describe a case of a 75-year-old man with renal cell cancer (RCC), who presented with secondary thrombocytopenia treated with steroid and IVIG. It is important to keep RCC in differential diagnosis for causes for secondary ITP as this impacts treatment.

## Introduction

Paraneoplastic syndromes are commonly associated with malignancy and occasionally can present as immune-mediated hematological syndromes. These include autoimmune hemolytic anemia (AIHA), the formation of anti-Factor VIII, and antiphospholipid antibody (APLA), along with immune thrombocytopenic purpura (ITP) among many others [1-3]. ITP leads to immune-mediated platelet destruction and suppressed production, and is one of the many causes for malignancy associated thrombocytopenia [4]. Herein, we present a case of a 75-year-old man who presented with secondary ITP in the setting of renal cell cancer (RCC).

## Case presentation

A 75-year-old Caucasian with a past medical history of chronic kidney disease (CKD) stage IIIa, Lyme disease, presented with one month of back pain, fatigue, and lower extremity swelling. In the emergency department, he was found to have a white blood cell (WBC) count of 12.5 K/uL, hemoglobin and hematocrit of 10 g/dL and 31.4 %, respectively (baseline of 14.9/45.9), mean corpuscular volume (MCV) of 83 fl, and platelet count of 2000 K/uL (previously normal). He reported easy skin bruising, eight-pound unintentional weight loss over the past few months along with chills. He denied tick bites, and home medications included aspirin 81 daily with multivitamin supplements. He denied smoking, alcohol, or drug use. 

The temperature was 37.2 Celsius, heart rate 85, blood pressure 124/62, and respiratory rate 20 on arrival. Neurological examination was normal. General examination was negative for hepatosplenomegaly, which, however, revealed petechia on lower extremities. Further workup revealed a reticulocyte index of 0.8 %. total bilirubin 0.6 mg/dL, aspartate transaminase (AST) 24U/L, alanine transaminase (ALT) 20U/L, and alkaline phosphatase (ALP) 141 U/L. Folate was >20.0 ng/ml, vitamin B-12 778 pg/ml, haptoglobin 451 mg/dL, iron 15 ug/dL , total iron-binding capacity (TIBC) 144ug/dl, ferritin 990ug/ dl, iron saturation 10 %, prothrombin time (PT) 18.8 sec, international normalized ratio (INR) 1.6, fibrinogen 693 mg/ dL, activated partial thromboplastin time (aPTT) was unavailable, and Lyme IgG/IgM levels were negative.

Peripheral smear was remarkable for thrombocytopenia, and red cell morphology was negative for parasites. Computed tomography (CT) of the head, liver function tests, human immunodeficiency virus (HIV), and Lyme antibody, Ehrlichiosis, Anaplasmosis, Babesiosis, and Hepatitis A, B, and C workup were all negative. Given the history of back pain, weight loss, and anemia, there was a concern for malignancy. CT abdomen was done that showed a large 8-cm hypervascular cystic and solid renal mass along with peritoneal adenopathy. Small bilobed hyperdense lesion in the right lobe of the liver was also observed. Findings were concerning for RCC.

**Figure 1:**
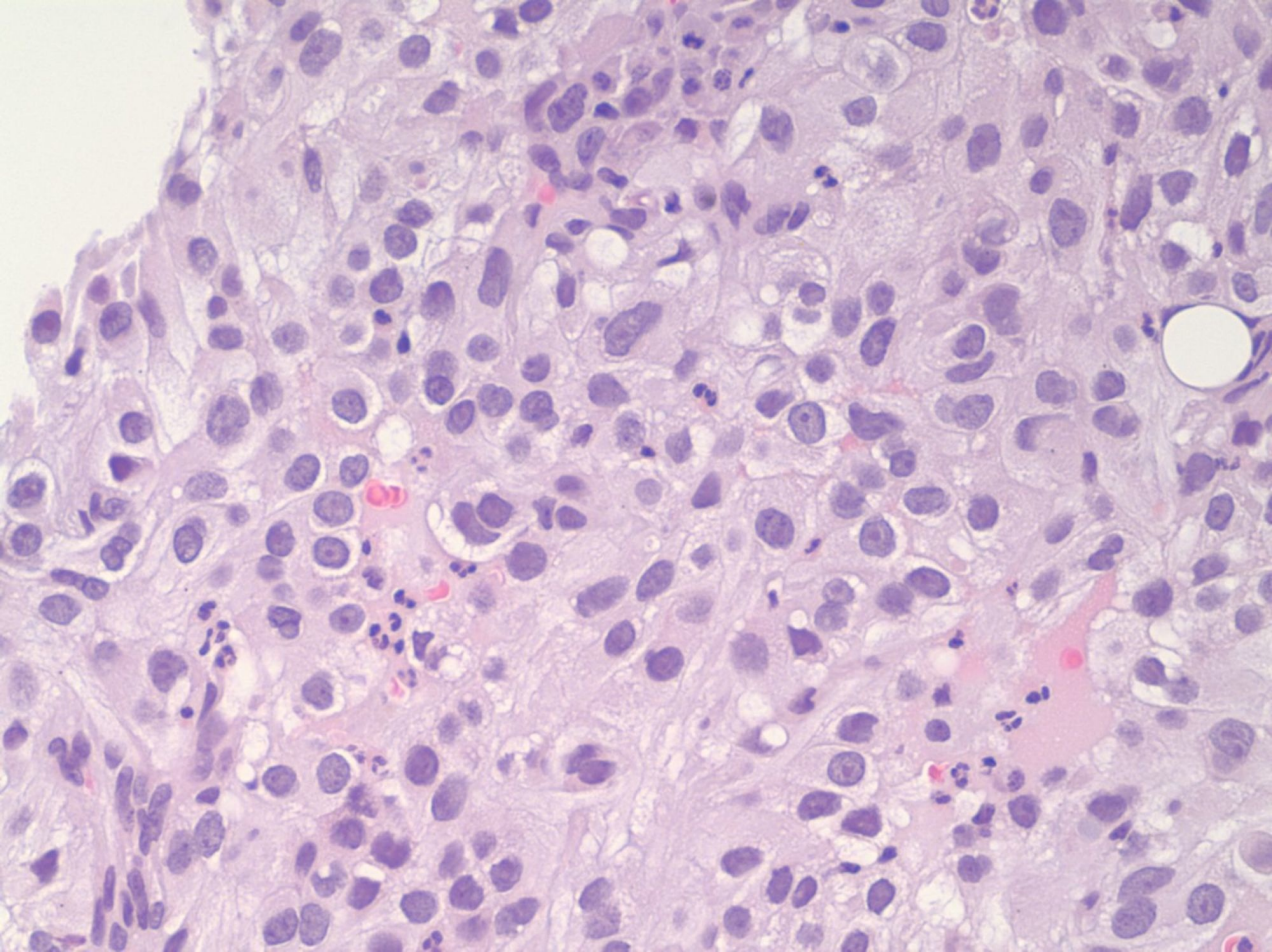
Solid sheet and nest of tumor occupying the bone marrow Pleomorphic and enlarged nuclei, delicate chromatin, visible nucleoli, and abundant eosinophilic cytoplasm can be observed.

**Figure 2:**
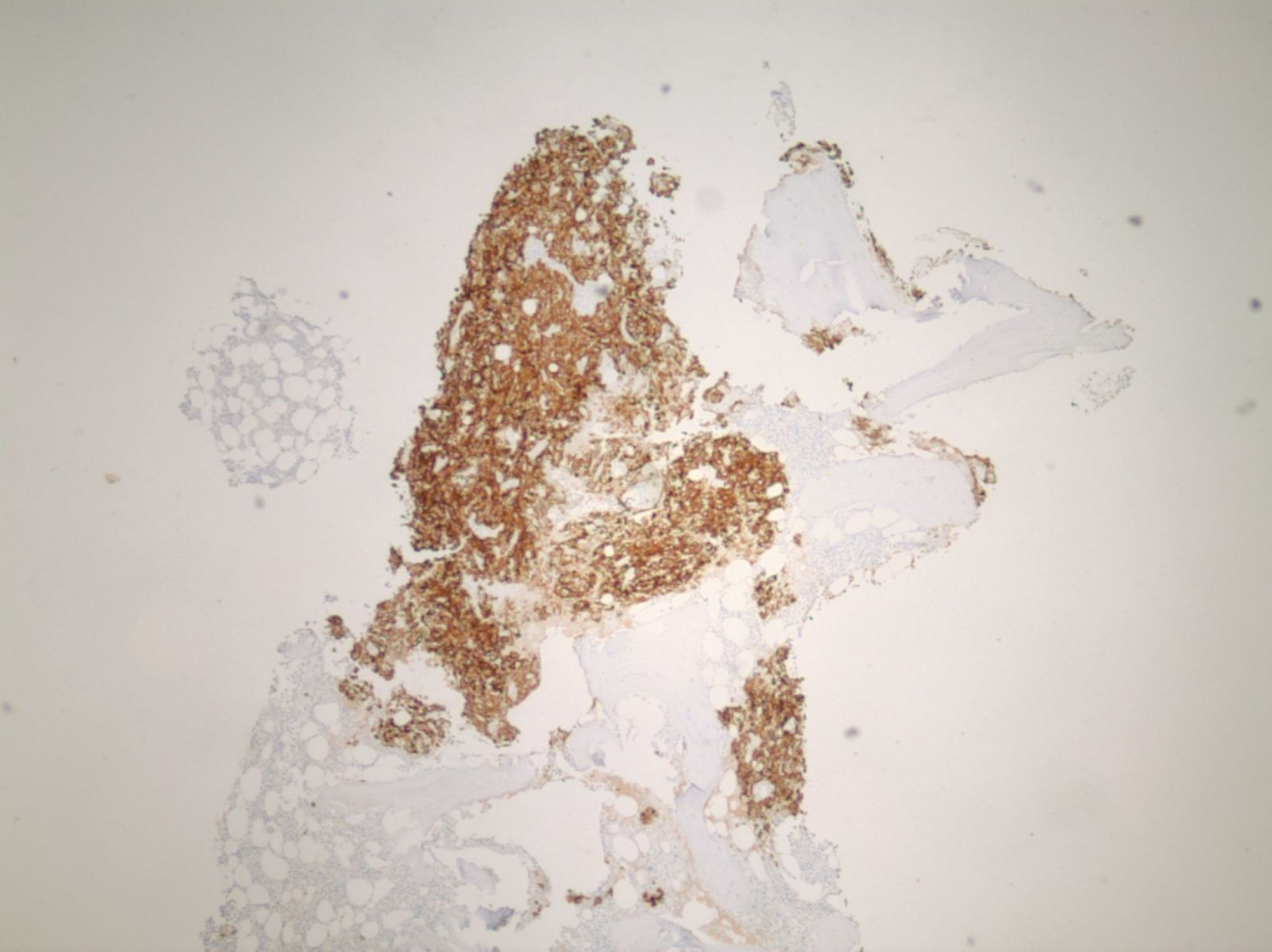
Immuno-histochemical stains AE1/AE3 compatible with renal cell carcinomas

**Figure 3:**
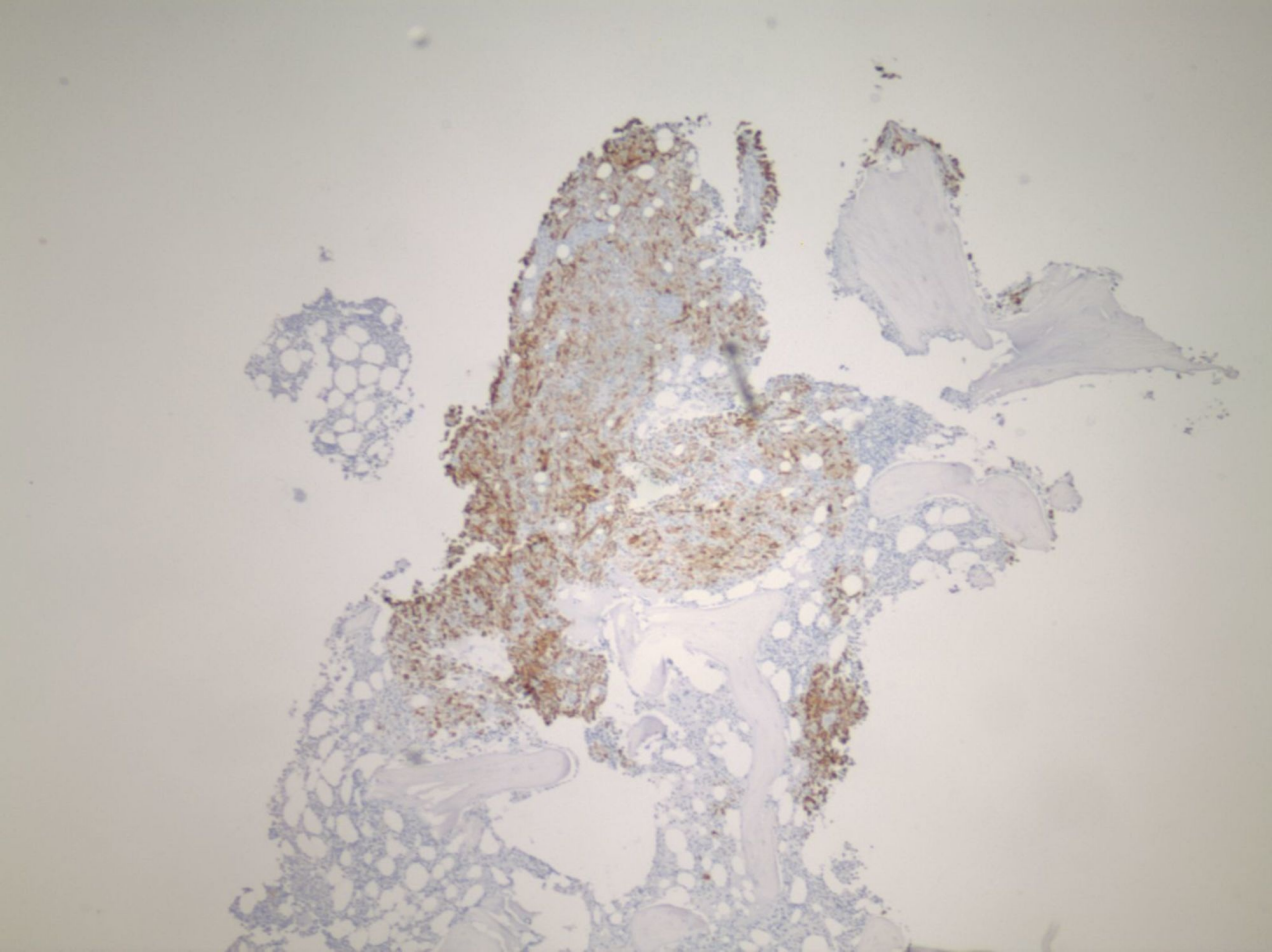
Immuno-histochemical stains CK7 compatible with renal cell carcinomas

**Figure 4:**
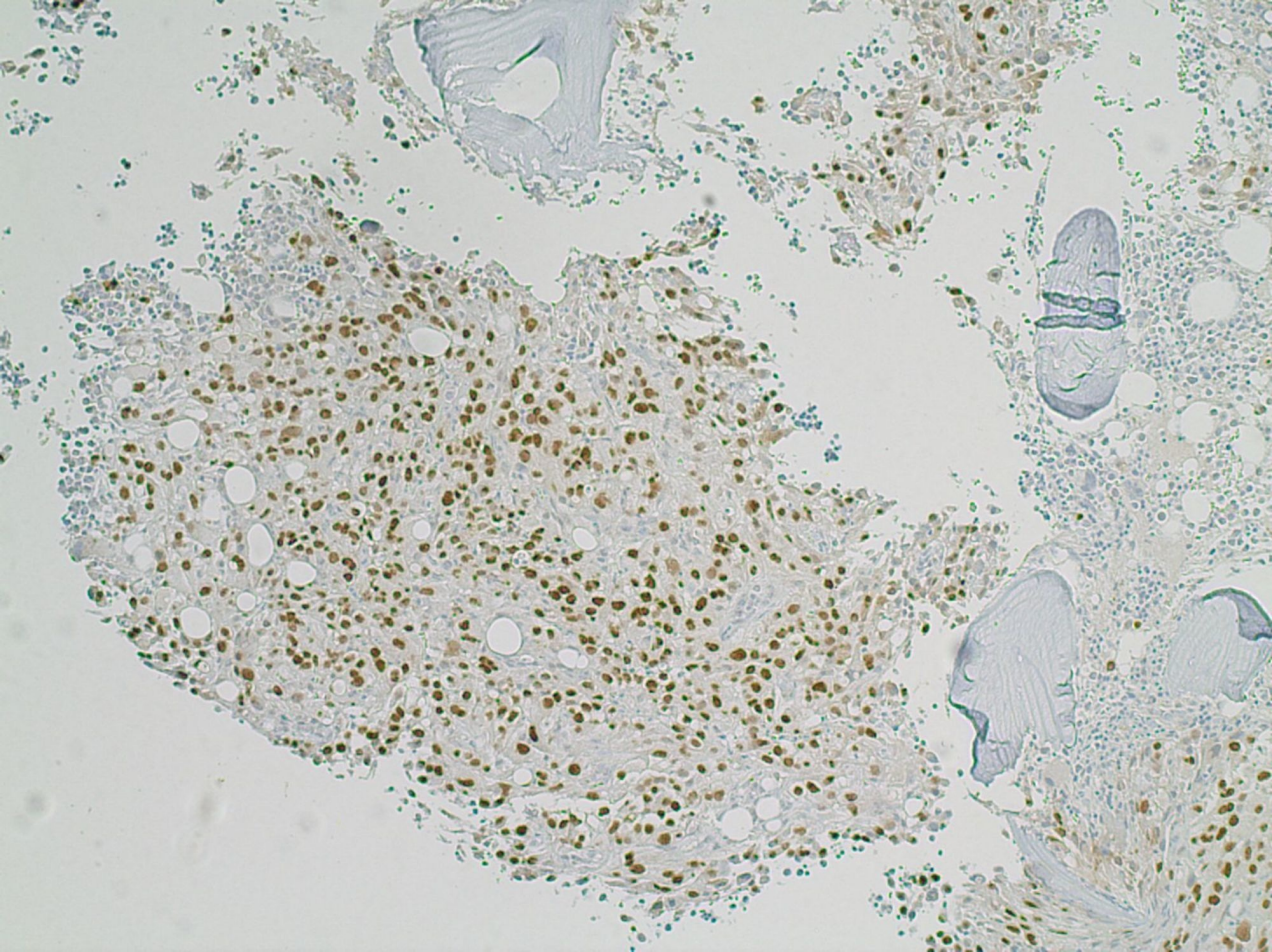
Immuno-histochemical stains PAX-8 compatible with renal cell carcinomas

The patient was started on intravenous immunoglobulin (IVIG) for five days along with prednisone given concern for renal cell cancer associated ITP. Bone scan was positive for the widespread metastatic osseous disease. Platelet count improved to 52 K/uL in a few days and the patient was discharged on oral steroids. He underwent bone marrow biopsy outpatient after his platelet count improved which showed metastatic carcinoma consistent with primary renal cell cancer, along with mildly hypercellular marrow and preserved trilineage hematopoiesis (Figures 1-4). Treatment was initiated with ipilimumab and nivolumab, which was poorly tolerated due to side effects. Due to the overall worsening clinical picture, he was made comfort measures only within four months of diagnosis.

## Discussion

Secondary ITP has been attributed to multiple etiologies such as infection, autoimmune disorders, vaccinations, medications, and lymphoproliferative disorders. 

ITP is reported in approximately two per 100,000 adults. It has been defined as isolated thrombocytopenia (platelet count <150,000 u/L) without an underlying illness [5]. Immune-mediated thrombocytopenia as a manifestation of paraneoplastic syndrome is a rare entity. If it does occur, it is typically seen with hematologic malignancies. Association with solid tumors appears to be rare but amongst the ones recognized, breast and lung cancer appear to be the most common. The least common occurrence is with renal cell carcinoma but the association has been identified [6].

Multiple theories have been postulated to explain thrombocytopenia in the setting of active malignancy. These include marrow aplasia due to chemotherapy and radiation, bone marrow infiltration by the tumor, drug-induced thrombocytopenia, disseminated intravascular coagulation, thrombotic thrombocytopenic purpura and hypersplenism resulting in widespread platelet consumption or overall immune dysregulation resulting in the destruction of platelets [6-10].

ITP has been described as first presenting manifestation of an underlying malignancy and in the majority of cases, secondary ITP occurs concurrently at the time of cancer diagnosis [11]. There have been case reports of ITP being diagnosed prior to the diagnosis of breast cancer and in some cases, it has been a presenting sign of cancer recurrence [12]. 

Renal cell carcinoma accounts for 2-3% of all cancers and 90% of all kidney malignancies [13]. It appears to be increasingly prevalent in Western countries with 50% being incidentally diagnosed on abdominal ultrasound or CT scan performed for various reasons. The disease burden is significant given a recent annual increase of nearly 2% in incidence worldwide and in Europe [14].

Various treatment options have been reported in the literature. The first-line approach includes the use of steroids, IVIG, either alone or in combination, along with resection of malignancy. This often leads to improvement in thrombocytopenia. Sustained complete remission has been reported following surgical resection alone, while sustained remission has been described following splenectomy together in combination with surgical resection of malignancy [15,16]. In certain cases, danazol, when tried prior to tumor resection in the patient with RCC, led to improvement in platelet counts [17].

Everolimus showed promise in a case report published in New South Wales, Australia for a patient with ITP refractory to oral and pulse dose steroids as well as IVIG. Although in prior trails, everolimus has resulted in thrombocytopenia, in that particular case, the use of everolimus showed sustained improvement in platelet count. That was likely due to immunomodulation and tumor lysis, indicating that addressing the underlying malignancy is imperative for treatment for this form of secondary ITP [7].

Given repeated observations of resolution of thrombocytopenia with the treatment of the underlying malignancy, localizing a possible underlying solid tumor is now considered an important part of the diagnostic workup for persistent or chronic ITP per American Society of Hematology (ASH) guidelines. X-ray, ultrasound, computed tomography are the diagnostic tools of choice. There needs to be a high index of suspicion to screen patients with a new diagnosis of ITP for an underlying malignancy, especially if treatment with steroids and IVIG has proved unsuccessful [18].

## Conclusions

Our case reports a relatively rare phenomenon, i.e., secondary ITP occurring as a paraneoplastic syndrome in the setting of renal cancer. It is important to keep renal cell carcinoma in differentials, especially while working up underlying etiology and triggers for secondary ITP, as this changes management. Our patient was diagnosed with metastatic renal cell carcinoma, and his platelet counts improved after treatment with IVIG and steroids. There is a scarcity of data regarding management, except for a few case reports. More studies need to be carried out in order to investigate the most effective treatment option for malignancy-associated ITP in the near future. 
